# Progress in Research on Regulated Cell Death in Cerebral Ischaemic Injury After Cardiac Arrest

**DOI:** 10.1111/jcmm.70404

**Published:** 2025-02-12

**Authors:** Zumin Chen, Shuangwei Wang, Tian Shu, Senlin Xia, Yanmei He, Yanhan Yang

**Affiliations:** ^1^ Huzhou Central Hospital Fifth School of Clinical Medicine of Zhejiang Chinese Medical University Huzhou China; ^2^ Guangdong Engineering Technology Research Center of Emergency and Life Support Medical Equipment Ambulanc (Shenzhen) Tech. Co., Ltd. Shenzhen China; ^3^ Huzhou Central Hospital Affiliated Central Hospital of Huzhou University Huzhou China

**Keywords:** cerebral ischaemic injury, cuproptosis, mechanism, regulated cell death, therapy

## Abstract

Ischaemic damage to the brain is the main cause of brain injury after cardiac arrest. The current treatment focuses on early reperfusion, but reperfusion tends to cause reperfusion injury, which is a significant problem. Cell death is an irreversible and normal end to cell life, playing key roles in maintaining the homeostasis and development of multicellular organisms. To date, cell death can be classified into two categories: accidental cell death (ACD) and regulated cell death (RCD). Cell death plays an indispensable role in cerebral ischaemia injury. An increasing number of scholars are exploring the mechanisms and sites of cell death during targeted inhibition of cerebral ischaemia to treat cerebral ischaemia injury. In addition to the established cell death pathways, namely, the apoptosis, pyroptosis and necroptosis pathways, ferroptosis and cuproptosis pathways have been discovered. This article reviews the cell death pathways involved in ischaemic brain injury, discusses the roles played by these death modalities, and suggests therapeutic directions for future targeting of cell death sites.

## 1 Introduction

With the continuous improvement of people's living standards and the marked increase in the ageing population, the incidence of cardiovascular and cerebrovascular events is increasing every year. At present, the main cause of death worldwide is out‐of‐hospital cardiac arrest (OHCA) [1]. Even with prompt cardiopulmonary resuscitation, OHCA has a high fatality rate, with an OHCA rate of 35 cases per 100,000 population in Europe and a survival rate of only 7.6%; the survival rate is 6.8% in North America and 3% in Asia [2]. The cause is the failure of vital organs, such as the brain, heart, and lung, caused by ischaemic injury (II) after OHCA, among which brain injury is the most lethal.

**FIGURE 1:**
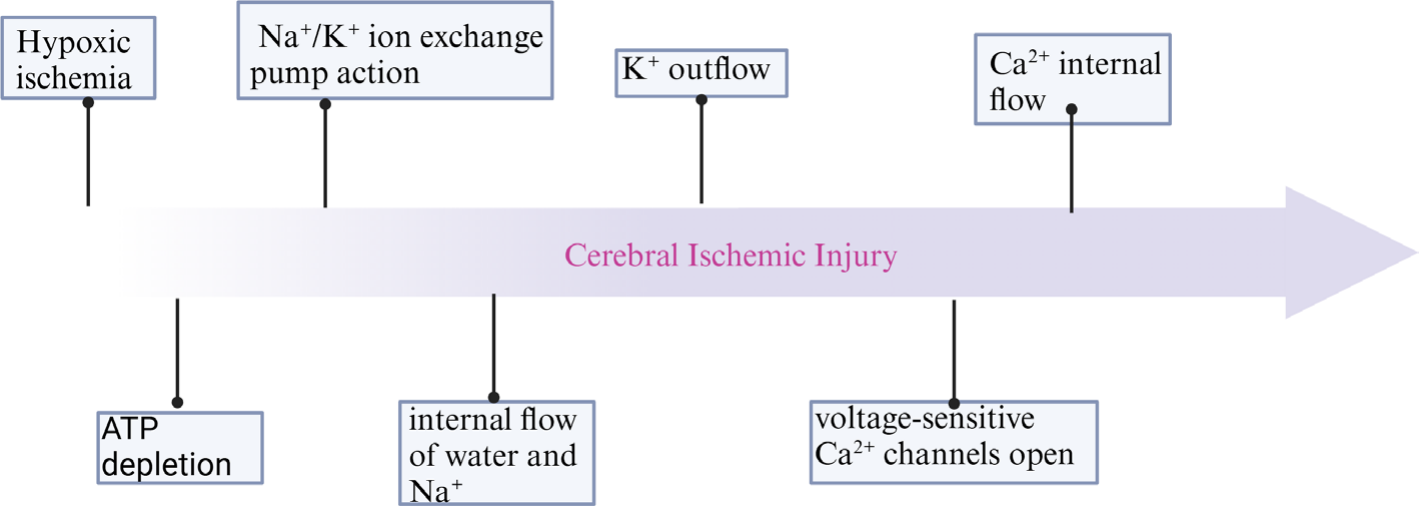
Following cerebral ischemia and hypoxia, brain activity is compromised, triggering a rapid onset of the ischemic cascade. The depletion of adenosine triphosphate (ATP) and glucose results in the dysfunction of the Na^+^/K^+^ ATPase pump, subsequently leading to mitochondrial injury and intracellular calcium overload. This cascade of events significantly exacerbates immediate cell necrosis or apoptosis.

When cardiac arrest occurs, the blood supply and oxygen supply to the whole body will stop within a few minutes [3], and the brain needs to consume 20%–30% of the cardiac output to maintain its function [4], so after the heart stops beating, the brain will also appear within a few minutes of neurogenic hypoglycaemia and metabolic crisis, resulting in nerve cell death. Within seconds of cerebral ischaemia and hypoxia, brain activity is impaired, and within minutes, an ischaemic cascade is rapidly set in motion, involving a series of biochemical events, including adenosine triphosphate and glucose consumption, Na^+^/K^+^ pump failure, and a loss of cellular structural integrity. These events then lead to mitochondrial damage and intracellular calcium overload, which further exacerbate immediate cell necrosis or apoptosis when levels of arachidonic acid, glutamate and other toxic excitatory neurotransmitters are elevated [5, 6, 7] (Figure 1). Clinically, the main manifestation of cerebral nerve injury after cardiac arrest is loss of consciousness, and the decrease in consciousness level after CA occurs within 20 s after the onset of ventricular fibrillation [8]. In observational studies, the loss of neurological function has been confirmed by isoelectric electroencephalography [9]. Due to the lack of inherent energy storage, neurons are particularly vulnerable to ischaemia, and cell damage begins immediately after a lack of cerebral blood flow (CBF). Clinical studies have shown that MRI shows signs of brain oedema during cardiac arrest and resuscitation [10].

Regulated cell death (RCD) plays an indispensable role in the mechanism of cerebral ischaemia injury. RCD is different from accidental cell death (ACD), which is not controlled via biological processes; in contrast, RCD is controlled by cell signalling cascades. Many different forms of RCD have been described, and they are all categorised by different mechanisms; frequently, more than one RCD pathway is simultaneously activated [11], and in some cases, elevated levels of one factor may indicate different activated cell death mechanisms. RCD has been extensively studied in many diseases; for example, copper cell death (cuproptosis) was discovered during cancer exploration [12], ferroptosis was discovered in studies of heart disease [13], apoptosis was first identified in research on infectious diseases [14] and pyroptosis was initially identified with respect to diabetic nephropathy [15]. At present, apoptosis, pyroptosis, necroptosis and ferroptosis of these RCDs have been found in ischaemic injury after cardiac arrest, which will be described in detail in the following article.

A variety of cell death modalities have been identified in cerebral ischaemia injury; therefore, studying the action sites and mechanistic pathways of cell death in cerebral ischaemia injury is important. In this paper, we discuss the mechanism of cerebral ischaemic injury due to various common types of RCD to summarise the mechanisms and potential targets of cell death modalities in cerebral ischaemic injury after cardiac arrest, thus providing ideas for the clinical treatment and prevention of cerebral ischaemic injury.

## 2 Mechanisms of Regulated Cell Death (RCD)

**FIGURE 2:**
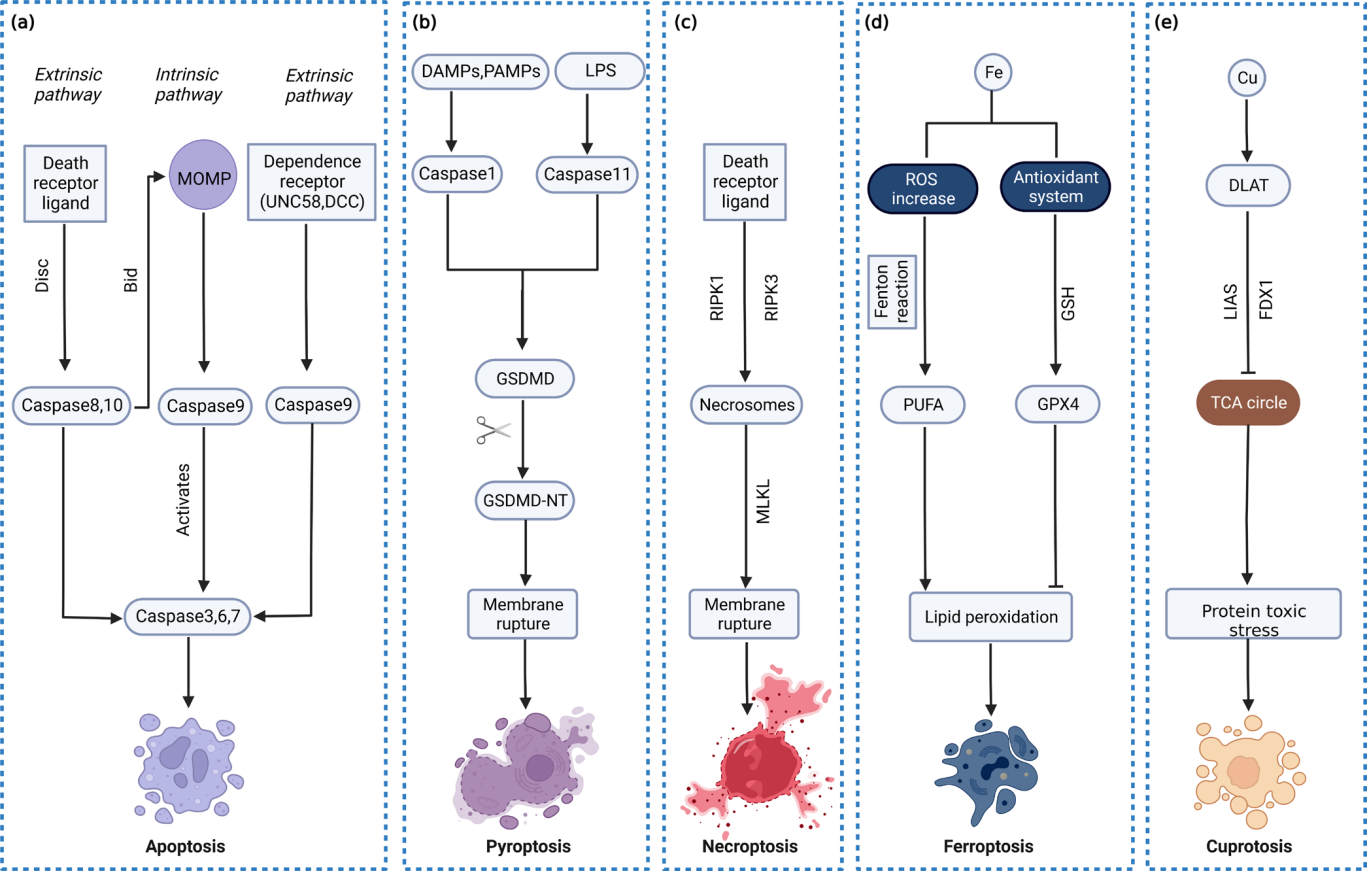
Apoptosis promotes cerebral ischaemic injury via Caspase3, Caspase6, Caspase7, Caspase8, Caspase10 and BCL2. Pyroptosis promotes cerebral ischaemic injury through GSDMD, Caspase1 and Caspase11. Necroptosis promotes cerebral ischaemic injury through RIPK3 and MLKL. Ferroptosis promotes cerebral ischaemic injury through ROX and GPX4. Cuproptosis promotes and inhibits cerebral ischaemic injury through FDX1.

As the cell biology field has evolved over the years, our understanding of the ways in which cells die has gradually improved, and the currently known cell death modalities include apoptosis, lysosomal cell death [8], pyroptosis, NETosis [16], necroptosis, entosis, parthanatos [17], ferroptosis, autosis [18], oxeiptosis and cuproptosis [19]. In this paper, five of the most common RCD mechanisms are selected to describe apoptosis, pyroptosis, necroptosis, ferroptosis and cuproptosis (Table 1; Figure 2).

**TABLE 1:** Mechanisms of regulated cell death.

PCD mode	Proposer	Mechanism	Initiating factors	Key factor	Reference
Apoptosis	Kerr	The active and orderly death of the body through gene regulation in order to maintain homeostasis.	Death receptor ligand, DNA damage	Caspase 3, Caspase 6, Caspase 7, Caspase 8, Caspase 10, BCL2	[13, 14, 15, 16, 17]
Pyroptosis	Cookson	Programmed death triggered by the inflammasome.	Inflammasome activation	GSDMD, Caspase 1, Caspase 11, IL‐18	[18, 19, 20, 21, 22, 23]
Necroptosis	Degterev	Death receptors are activated to induce the formation of necrosomes, which then lead to membrane rupture.	RIPK3 activation	RIPK3, MLKL, TLR	[24, 25, 26, 27, 28, 29, 30]
Ferroptosis	Dixon	Programmed cell death was driven by iron accumulation and lipid peroxidation.	Increased iron accumulation and lipid peroxidation	ROX, GPX4, Fe, GSH	[31, 32, 33, 34, 35]
Cuproptosis	Tsvetkov	The combination of copper ionophore and Fe‐s results in the inhibition of mitochondrial respiration and death.	Cu accumulation	FDX1, Cu, DLAT, LIAS	[12]

### 2.1 Apoptosis

The mechanism of apoptosis was first proposed by Kerr, Wyllie, and Currie in 1972 [20]. After decades of research, our understanding of apoptosis has increased, and it is now classified into two categories: extrinsic apoptosis and intrinsic apoptosis. Exogenous apoptosis is caused by the activation or deactivation of death receptor ligands and is driven by the initiators caspase 8 and caspase 10 [21], which are primarily responsible for the initiation of apoptotic pathways, and the effectors caspase 3, 6 and caspase 7, which are responsible for the explicit cleavage of cell components [22]. It is mediated by membrane receptors, particularly the Fas cell surface death receptor CD95 and the TNF receptor TNFR1. In addition, extrinsic apoptosis can be induced by activation of the dependent receptors UNC58 and DCC through the executor caspase9 or by the dephosphorylation of DAPK1 after disengagement from its ligand [23]. DNA damage, hypoxia, metabolic stress and other factors can induce intrinsic apoptosis because mitochondrial outer membrane permeability (MOMP) causes the release of mitochondrial proteins (such as cytochrome C and Smac.) that activate the executor caspase9, and pro‐casp9 is induced to form apoptotic bodies [24]. MOMP is regulated by proapoptotic factors such as BAX in the apoptosis‐regulating BCL2 family and antiapoptotic factors such as Bcl‐XL [25]. The extrinsic apoptosis pathway triggers intrinsic mitochondrial apoptosis by activating caspase8, which cleaves BID (TBID). TBID is then translocated to mitochondria to induce MOMP through the activation of BAX and BAK1. Caspase3, caspase6 and caspase7 are considered coeffector caspases of extrinsic and intrinsic apoptosis.

### 2.2 Pyroptosis

Pyroptosis was first defined by Fink et al. in 2001 [26]. Pyroptosis is triggered by an inflammasome, which is a cytosolic multiprotein complex critical for the release of interleukin (IL) 1 family members such as interleukin‐1β [IL1B] and IL18. Apoptosis‐associated CARD‐containing spot‐like protein (ASC, also known as PYCARD or PYRIN‐ and CARD‐domain proteins) specks are formed, and RCD is driven by proinflammatory caspases [27]. Recent studies have shown that Gasdermin D (GSDMD) is a key effector of fever‐inducing spirochetes [28]. GSDMD is cleaved by caspase11 or caspase1 [29], and the self‐cleavage of caspase11 at an intersubunit link is particularly significant for the cleavage of GSDMD [30]. After cleavage, GSDMD‐N is transferred to the inner leaflet of the plasma membrane, where it binds to phospholipids, inducing the formation of pores and ultimately causing membrane lysis [31]. This whole process is known as pyroptosis.

### 2.3 Necroptosis

Necroptosis is a new term put forward by Degterev in 2005 [32]; it is a form of programmed necrosis similar to apoptosis, but in contrast to apoptosis and other forms of programmed cell death, necroptosis does not depend on caspase activity [33]. When caspase8 activation of the death‐inducible signalling complex (DISC) is inhibited, the same ligands, such as TNFSF10 and the ligand FASLG, can trigger necrosis via the extrinsic apoptotic pathway [34]. Subsequent studies have proven that threonine kinase 3 (RIPK3) is a key factor in necrotizing apoptosis [35] and that mixed lineage kinase domain‐like pseudokinase (MLKL) is an effector of necroptosis [36]. First, the death receptor ligand induces RHIM‐mediated RIPK1 binding to RIPK3, triggering the formation of specific signalling complexes known as necrotic bodies and leading to MLKL phosphorylation [37]. The phosphorylation of MLKL by RIPK3 results in a conformational change. Then, inositol hexaphosphate (IP6) binds to a positively charged region in the N‐terminus of MLKL, enabling its recruitment to a phosphatidylinositol site. MLKL‐N then inserts into the plasma membrane and polymerises, leading to membrane rupture [38]. This chain reaction is called necroptosis.

### 2.4 Ferroptosis

Ferroptosis was first proposed by Dixon in 2012 [39]. It has two ways of inducing cell death. In one mechanism, a classic pathway is triggered by the inactivation of GPX4, which is the main factor protecting biofilms against peroxidation damage. The other mechanism involves an atypical increase in the unstable iron pool. The increase in the iron pool leads to the accumulation of iron in the body, and in cells, large quantities of divalent iron accumulate. The generation of ROS from divalent iron by the Fenton reaction leads to ferroptosis [40]. In addition, iron‐dependent lipid reactive oxygen species (L‐ROS) formation, glutathione (GSH) depletion and lipid repair enzyme peroxidase 4 (GPX4) inactivation are involved [41]. The peroxidation of polyunsaturated fatty acids (PUFAs) catalysed by iron leads to the abnormal accumulation of L‐ROS, leading to membrane rupture and subsequent cell death. The main target of membrane lipid peroxidation in the ferroptosis pathway is PUFAS [42]. This harmful effect of lipid peroxidation can be neutralised by lipophilic free radicals, such as vitamin E [43]. Ferroptosis is simply considered caused by an imbalance between ROS generation induced by iron accumulation and the antioxidant system activated during lipid peroxidation, and GPX4 is considered a key marker of ferroptosis.

### 2.5 Cuproptosis

Cuproptosis is a form of cell death discovered by Tsvetkov et al. [19]; it differs from apoptosis, ferroptosis, pyroptosis and necroptosis, which have been previously described. Tsvetov called the cell death mechanism that they identified cuproptosis. Moreover, this scientist found that cells that rely on mitochondrial respiration are approximately 1000‐fold more sensitive to cuproptosis inducers than cells that rely on glycolysis. Inhibitors of electron transport chain (ETC) complexes and mitochondrial pyruvate uptake both reduce the cuproptosis rate. The content of metabolites associated with the tricarboxylic acid cycle (TCA) is changed in cells treated with copper ionophores. According to studies performed to date, cuproptosis may act in conjunction with the TCA. Recently, genome‐wide CRISPR/Cas9 knockout screening combined with metabolic and biochemical assays led to the identification of two mitochondrial proteotoxic stress pathways mediating cuproptosis. Notably, copper was associated with an increase in mitochondrial protein acylation. In addition, mass spectrometry (MS) proteomics showed that the FDX1‐dependent degradation of Fe–S cluster proteins may contribute to cuproptosis. At the end of this experiment, the authors investigated whether natural copper without the oxidative stress inducer elesclomol (ES) induces outcomes similar to those induced by ES and copper. The addition of CuCl2 preferentially resulted in a cuproptosis phenotype in cells overexpressing the copper transporter solute carrier family 31 member 1 (SLC31A1). Depletion of glutathione by BSO, a potent inhibitor of γ‐glutamylcysteine synthetase, also increased the susceptibility of A549 lung cancer cells to cuproptosis. Aged ATP7B‐deficient mice (Atpb7b−/− used to establish a model of Wilson's disease, a copper dysregulation syndrome) were compared with heterozygous (Atpb7b+/−) and wild‐type control mice. The atp7b‐deficient mice presented with significantly lower levels of thiocyanated protein and Fe–S cluster protein in the liver, and the level of the hsp70 protein was significantly increased. Similar to ATP7B, ATP7A is also involved in the copper death process, and the related diseases it affects have three manifestations: Menkes disease (MNK), occipital horn syndrome (OHS) and X‐linked distal spinal muscular atrophy 3 (SMAX3) [44]. ATP7A, a homologous P‐type ATPase with ATP7B, uses energy to pump copper across the membrane. ATP7A moves copper out of the cytoplasm of extrahepatic tissue and across the basolateral membrane [45], while ATP7B moves copper out of the cytoplasm and across the top membranes of the liver, brain, and kidney [46]. Various models have proven that cell death induced by copper homeostasis is the same mechanism as cell death induced by copper ionophores. The core molecule of cuproptosis is currently believed to be FDX1, which can reduce Cu^2+^ to Cu^1+^, which is more toxic, when copper accumulates and catalyses the fatty acylation of DLAT, DLST and LIST. Cu^1+^ further binds to the fatty acylation site, resulting in oligomyelization of DLAT, thus inducing copper toxicity [19]. In summary, the main process of copper‐related cell death depends on the accumulation of intracellular copper ions, which bind directly to the acylated components of the TCA cycle, resulting in the aggregation and dysregulation of these proteins, which blocks the TCA cycle, triggering proteotoxic stress and induces cell death.

## 3 Role of RCD in Cerebral Ischaemic Injury

**FIGURE 3:**
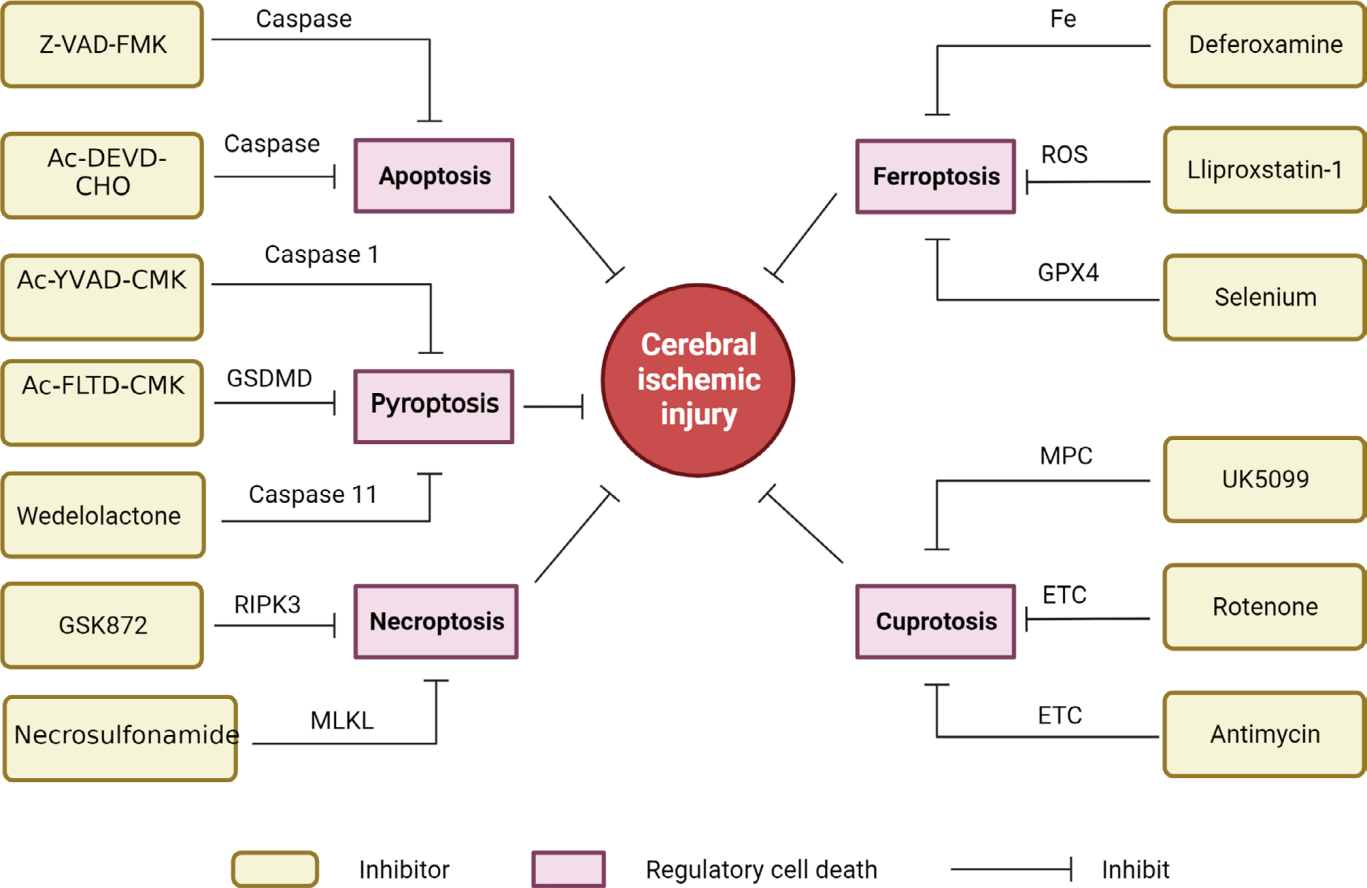
VAD‐FMK and Ac‐DEVD‐CHO protect against cerebral ischaemic injury by inhibiting caspase, thereby inhibiting apoptosis. Ac‐YVAD‐cmk inhibits cerebral ischaemic injury by inhibiting caspase1 activity, Ac‐FLTD‐CMK inhibits GSDMD activity, and wedelolactone inhibits pyroptosis by inhibiting caspase11. GSK872 affects necroptosis by inhibiting RIPK3 and necrosulfonamide by inhibiting MLKL function; therefore, they may confer protection against cerebral ischaemic injury. Deferoxamine inhibits Fe ion production, liproxstatin‐1 inhibits ROS production, and selenium inhibits GPX4 activity, all of which inhibit ferroptosis and thus protect against ischaemic brain injury. UK5099 inhibits MPC, and rotenone and antimycin inhibit the ETC, suppressing cuproptosis and thus preventing cerebral ischaemic injury.

After cardiac arrest, blood flow to the brain is largely blocked; thus, the brain is damaged by ischaemia, and the mechanisms of ischaemic injury are complex. Inadequate oxygen delivery during ischaemia leads to disruption of ion pump function, so intracellular Ca^2+^ overload is detected, and continuous Ca^2+^ overload can lead to ROS overproduction beyond physiological thresholds and further disruption of mitochondrial function [47], including the continued opening of permeability transition pores and MIM depolarization, ultimately leading to cell death [48]. Oxidative stress is caused by oxidative damage induced by an imbalance of reactive oxygen species (ROS) generation and consumption [49] and leads to oxygen production mediated via free radicals by activating the oxidative stress response, which causes mitochondrial damage and activates protein kinases, causing brain nerve death [50]. Thus, oxidative stress activates cytokine receptors and inflammatory pathways involved in delayed neuronal damage after hypoxia, such as acidosis and cell death, but oxidative stress is also a major mechanism associated with reperfusion [51]. This review is focused on the relationship between several mechanisms of cell death and cerebral ischaemia injury (Figure 3).

### 3.1 Apoptosis and Cerebral Ischaemic Injury

Apoptosis is considered the key to ischaemic brain injury [52]. Many researchers have confirmed this supposition via experiments. For example, He et al. [53] identified apoptosis via flow cytometry, showing a significant increase in apoptotic cells in a model of brain ischaemia induced by oxygen and glucose deprivation (OGD) compared to the number in the control model. A Western blot analysis showed that the apoptosis‐related proteins caspase3 and caspase8 were significantly overexpressed in the OGD group. In another study, Li et al. [54] found that the protein expression of BAX was significantly increased in rats with ischaemic injury. In Anna Kleczka's study [55], it was found that the expression of the proapoptotic BAX gene and antiapoptotic BCL2 gene in ovarian OV7 cancer cells treated with caffeic acid phenethyl ester (CAPE) was significantly higher than that in the control group. The results could be obtained after evaluating the slope (gradient) analysis of BAX and BCL2. The slope value of proapoptotic BAX expression was 10 times that of antiapoptotic BCL2 expression. This indicates that the increase in the BAX/BCL2 ratio can significantly promote the pace of apoptosis. In Abrizian's study [56], it was found that the protective effect of neuroprotection on ischaemic brain injury was produced by reducing the expression level of BAX/BCL2. The BAX, BAX/BCL2 ratio, caspase3 and caspase8 protein levels are markers of apoptosis, and the experimental results showed that apoptosis promoted the progression of ischaemic injury.

### 3.2 Pyroptosis and Cerebral Ischaemic Injury

Recent reports have suggested that pyroptosis is involved in a wide range of central nervous system diseases, including cerebral ischaemia injury and ischaemia–reperfusion injury [57]. Zhao et al. [58] found that inhibition of caspase1 induced antiapoptotic processes and attenuated neuronal damage in the hippocampus after cerebral infarction. Yi Zhou [59] found that the expression of the pyroptosis factor caspase1 was significantly increased in the ischaemic injury group on the basis of protein imprinting in mice with cerebral ischaemia injury. In addition, the expression of GSDMD, a key pyroptosis factor, was increased significantly in the ischaemic injury group. Ran Y [60] showed increased expression of caspase1 and GSDMD using in vitro models. Because of the important role of caspase1 and GSDMD in pyroptosis, the abovementioned experiments showed that pyroptosis plays an important positive role in cerebral ischaemic injury.

### 3.3 Necroptosis and Ischaemic Brain Injury

Increasing evidence shows that necroptosis plays an important role in cerebral ischaemic injury [32] and that inhibition of necroptosis can reduce the size of infarct caused by cerebral ischaemic injury [61]. Li et al. [62] simulated ischaemic damage to isolated hippocampal neurons, and Western blotting showed that the level of RIPK3 in the ischaemic group was significantly increased compared with that in the untreated group. Li subsequently found similar results with a mouse model, in which the expression levels of RIPK3 and MLKL were significantly increased in a middle cerebral artery occlusion (MCAO) model group. Chen et al. [63] found that the expression levels of p‐RIP1/RIP1 and p‐MLKL/MLKL, the activation markers of necrotic apoptosis in the area around cerebral infarction in tMCAO rats, gradually increased from the first day and reached the highest level on the third day. Deng, Li, and Sun [64] found that after Necrostatin‐1 inhibited the RIPK1‐mediated RIPK3/MLKL signalling pathway, the infarct volume of brain tissue significantly decreased, and the neural function score significantly increased. Since RIPK3 is the key factor in necroptosis and MLKL is an important effector, necroptosis plays a significant role in promoting cerebral ischaemia injury.

### 3.4 Ferroptosis and Cerebral Ischaemic Injury

Recently, many scholars have found that iron plays an indispensable role in cerebral ischaemic injury [65] and that the higher the content of serum iron is, the greater the probability of poor prognosis [66]. Tuo [67] found a marked increase in the iron content in the lesioned hemisphere in a rat model of MCAO; then, after these rats were administered ferrostatin‐1, a ferroptosis inhibitor, the cerebral infarct size of the rats decreased significantly compared with that in the control rats, indicating that inhibition of ferroptosis significantly reduced ischaemic injury. Lu, Xu, and Lu, [68] found that the accumulation of free iron and ROS in brain tissue increased significantly and that the levels of GPX47 and GSH decreased significantly. In this study, Li et al. [69] used electroacupuncture amendments (EA) to treat NCAO rats and found that EA could significantly improve the activity of GPX4 and SOD as well as the level of GSH and reduce the accumulation of MDA and iron to reduce the level of ischaemic injury in brain tissue by inhibiting ferroptosis. These results indicate that ferroptosis plays a very important role in brain injury and that inhibiting iron‐related death can exert an excellent protective effect on the brain.

### 3.5 Cuproptosis and Cerebral Ischaemic Injury

Cuproptosis is a recently discovered form of RCD, and everything about this modality is still being explored. Jiang et al. [70] showed that chronically ingested copper in the form of copper sulfate in drinking water increased free copper levels, which exerted a toxic effect on the brain. Another study by Lai et al. [71] found that serum levels of total copper and loosely bound small molecules containing copper were increased in patients with ischaemic stroke. Lai also found that the levels of total serum copper, urine copper, and small‐molecule copper (SMC) complexes in patients with ischaemic stroke were higher than those in the control group. The plasma ceruloplasmin (CP) concentration was not significantly different between groups, but its activity was increased by 59% in the high‐copper groups. The homocysteine (HCY) concentration was 33% higher than that of the control group. To investigate this outcome, Lai performed an association analysis and found that the SMC complex concentration was positively correlated with total copper in urine, Hcy concentration in serum, and CP activity. The activity of CP in serum in the presence or absence of copper ions was determined by zymography. Copper ions improved the activity of CP, which is an antioxidant that protects against oxidative stress, but in contrast, CP oxidises LDL, which in turn promotes ROS production [72]. Lai showed that elevated CP activity, not increased CP concentration, was associated with an increased risk of ischaemic stroke. Studies by Altamura et al. [73] and Kodali et al. [74] supported Lai's experimental results; thus, a relationship between cerebral ischaemic injury and copper‐related death can be conclusively assumed. Huusokonen et al. [75] found that the copper dithiocarbamate complex CuII (atsm) protected neurons from excitotoxic effects in vitro. N2a cells were protected from OGD‐induced injury in an in vitro brain ischaemia model, and CuII (atsm) showed protective effects in permanent and transient mouse models of ischaemia, as determined by functional results and damaged area measurements. In addition, copper–zinc superoxide dismutase (SOD1) reduced the level of mitochondrial cytochrome c release and the degree of subsequent caspase activation. These results suggested that SOD1 exerted a protective effect on neuronal injury after global cerebral ischaemia [76]. All this evidence showed that copper‐induced death exerted a complex effect on cerebral ischaemic injury. Cuproptosis not only accelerated and aggravated the generation of injury but also protected tissues through certain mechanisms. The reasons for these outcomes and the mechanisms involved are worthy of further exploration.

### 3.6 Interaction of Different RCDs in the Context of Cerebral Ischaemia

Despite great differences in the pathways and characteristics among the abovementioned RCDs, there is still much crosstalk between the regulation of various RCDs. First, a large number of lipid peroxidation products that are produced in the process of ferroptosis can induce oxidative stress, which leads to necrosis, apoptosis and other RCDs. During ferroptosis, energy metabolism is often abnormal and is accompanied by mitochondrial size reduction, mitochondrial ridge reduction and increases in the mitochondrial membrane density and mitochondrial membrane rupture, which suggests that there may be some crosstalk between ferroptosis and mitochondrial autophagy [39]. At the same time, owing to the abnormal metabolism of arachidonic acid and inflammation in the process of ferroptosis, there may be some crosstalk between ferroptosis and an inflammatory RCD. Second, in the process of pyroptosis, as a kind of inflammatory cell death, the inflammatory factors produced can activate the downstream NF‐κB pathway, triggering a proinflammatory mechanism, apoptosis, and necroptosis [77]. At the same time, overactivated mitochondrial autophagy leads to cell energy metabolism disorders and induces an imbalance in internal and external ion regulation, which results in ferroptosis, cuproptosis and other forms of RCD [78]. The crosstalk among pyroptosis, apoptosis and necroptosis indicates the presence of a dynamic molecular interaction network, which has been conceptualised as PANoptosis and represents an inflammatory RCD that is activated by specific triggers and has molecular characteristics of pyroptosis, apoptosis, and necroptosis [79].

## 4 Basic Experimental and Clinical Applications

**FIGURE 4:**
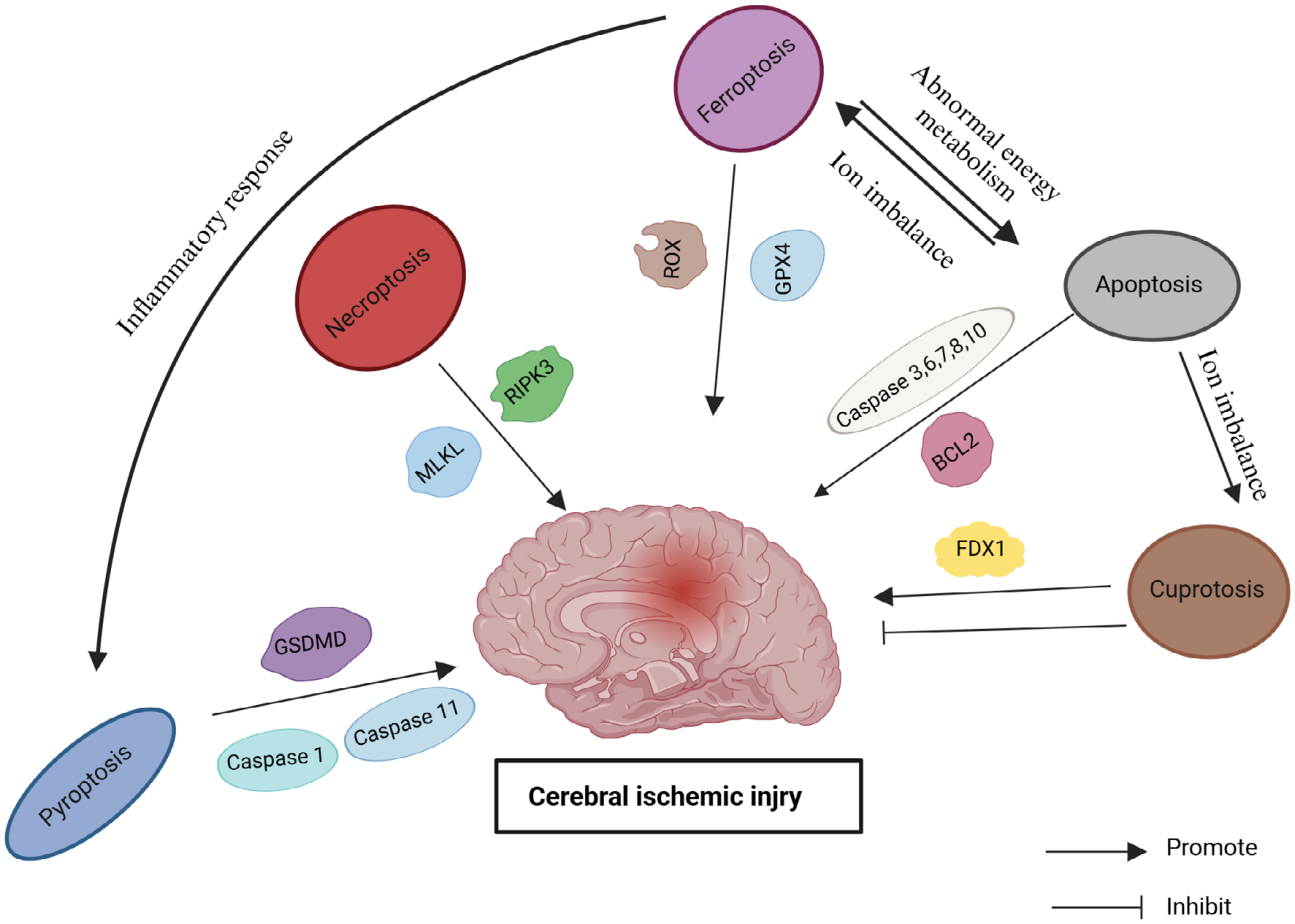
The central mechanism of regulatory cell death. Apoptosis is classified into extrinsic and intrinsic apoptosis. Extrinsic apoptosis is caused by the binding or dissociation of death receptor ligands and their receptor ligands. DNA damage, hypoxia, metabolic stress and other factors can induce extrinsic apoptosis. Caspase1 and Caspase11 lead to GSDMD cleavage, and the cleavage products bind to phospholipids, resulting in membrane cleavage. A ligand binds to a death‐inducing complex to form necrosomes, which undergo conformational changes resulting in membrane rupture. Ligand–death receptor binding also promotes the ROS production system by increasing iron levels and inhibits the antioxidant system causing lipid peroxidation, leading to ferroptosis. The accumulation of intracellular copper ions, which directly bind to acylated lipid components in the TCA cycle, blocks the TCA cycle, triggers proteotoxic stress, and induces cell death.

At present, there is no direct treatment for cerebral ischaemic injury after cardiac arrest, so secondary damage to the brain should be minimised by maintaining physiological homeostasis. Disturbance of body temperature, arterial blood pressure, oxygenation and ventilation should be avoided [80]. Many treatments are applied for ischaemic brain injury after cardiac arrest, such as hypothermia, in which a patient is induced into a hypothermic state at the time of treatment [81]. In the HYPERION trial [82], they enrolled 581 patients with non‐shock rhythm (sudden arrest or pulse less electrical activity) who were resuscitated from OHCA or in‐hospital cardiac arrest (IHCA) to TTM at 33°C and 37°C and found that the rate of favourable neurological outcomes was significantly higher in the hypothermia group than in the non‐hypothermia group, but there was no significant improvement in survival. A 2021 trial [83] even found that hypothermia increased the risk of arrhythmia and haemodynamic instability. Therefore, The European Resuscitation Council (ERC) and the European Society of Intensive Care Medicine (ESICM) have updated their guidelines to avoid fever (> 37.7°C) for at least 72 h after ROSC [80]. ERC guidelines for resuscitation care after cardiac arrest include ventilation, temperature control, oxygenation, reperfusion and critical monitoring, but there are no specific medication guidelines. However, early reperfusion of CBF is the most recognised treatment. Both of these treatments show certain drawbacks. Hypothermia may cause other problems due to low body temperature, and reperfusion therapy often leads to reperfusion injury. To prevent these outcomes, whether it is possible to target the mechanisms of ischaemic injury and use drugs to prevent these injuries is still unknown. The abovementioned mechanisms of cell death have an impact on the occurrence and development of cerebral ischaemia injury; therefore, these mechanisms may show corresponding applications to experimental exploration and clinical treatment (Table 2; Figure 4). Despite the recent publication of various reports on novel RCD activators and inhibitors, clinical trials evaluating the effects of novel RCD modulators are still in their infancy.

**TABLE 2:** Basic experimental application and clinical application.

PCD mode	Major inhibitors (target)	Basic application	Clinical application	Progress of cerebral ischaemic injury	Reference
Apoptosis	Z‐VAD‐FMK, Ac‐DEVD‐CHO (caspase)	Rab27a prevents apoptosis by downregulating cleaved caspase‐3 expression.	ZNS can inhibit neuronal apoptosis in cerebral ischaemia.	Active	[40, 60, 61, 62]
Pyroptosis	Ac‐YVAD‐cmk (caspase 1), Ac‐FLTD‐CMK (GSDMD), wedelolactone (caspase 11)	CHRFAM7A overexpression can inhibit pyroptosis through NLRP3/Caspase‐1 dependent pathway.	Remimazolam can inhibit cell pyroptosis by inhibiting NLRP3.	Active	[63, 64, 65]
Necroptosis	GSK872 (RIPK3), necrosulfonamide (MLKL)	TRAF2 inhibits necroptosis by reducing the association between MLKL and RIP3.	Ponatinib combined with emricasan can inhibit the necrotizing apoptosis of brain injury by inhibiting the combined pathway of caspase8 and RIPK3.	Active	[47, 66, 67]
Ferroptosis	Deferoxamine (Fe), liproxstatin‐1 (ROS), selenium (GPX4)	Selenium inhibits ferroptosis in part by inhibiting GPX4.	Sulfasalazine can induce the occurrence of iron death to treat pancreatic cancer.	Active	[31, 68, 69]
Cuproptosis	UK5099 (MPC), rotenone and antimycin (ETC)	The MPC inhibitor UK5099 and ETC complex I/III inhibitors attenuate elesclomol‐induced cuproptosis.	Elesclomol for melanoma	Negative and active	[70, 71, 72, 73]

### 4.1 Apoptosis in the Treatment of Cerebral Ischaemic Injury

According to the aforementioned mechanism, extrinsic apoptosis is controlled by caspase8 and caspase10 activity, and intrinsic apoptosis is caused by MOMP. While MOMP is controlled by the BCL2 protein family, caspase3, caspase6 and caspase7 are effectors in both extrinsic and intrinsic apoptosis. Therefore, caspase inhibitors (Z‐VAD‐FMK, Ac‐DEVD‐CHO, etc.) [84] can effectively inhibit apoptosis. In basic experiments, many drugs have been verified to inhibit apoptosis, thereby reducing cerebral ischaemic injury. *Carthamus tinctorius* L. was shown by Zhang [85] to attenuate apoptosis after cerebral ischaemic injury by inhibiting Bax and caspase‐3 activation and increasing Bcl‐2 expression levels. Ma found that Rab27a controlled the secretion and function of exa in the brain, preventing apoptosis by downregulating the cleavage of caspase‐3, and thereby protecting cerebral blood vessels from ischaemic injury. Some drugs, such as zonisamide (ZNS), might be used in the clinic. Condello [86] used ZNS to preserve mitochondrial function and block apoptotic signalling pathways. Based on Condello's work, He et al. [53] investigated the effect of ZNS on the nervous system and found that ZNS inhibited the apoptosis of nerve cells during cerebral ischaemia, showing great promise for clinical application.

### 4.2 Pyroptosis in the Treatment of Cerebral Ischaemic Injury

The key factor in pyroptosis is GSDMD, which is controlled by caspase11 and caspase1. Therefore, Ac‐YVAD‐cmk was targeted to inhibit caspase1, wedelolactone was targeted to inhibit caspase11, and Ac‐FLTD‐CMK was targeted to affect GSDMD, inhibiting pyroptosis. CHRFAM7A [87] overexpression inhibited pyroptosis by influencing the NLRP3/Caspase1‐dependent pathway, thereby conferring protection against cerebral ischaemic injury. In Huang's study [88], curcumin inhibited NLRP1‐dependent neuronal apoptosis to protect against cerebral ischaemic injury. Shi [89] demonstrated that remimazolam inhibited pyroptosis by inhibiting NLRP3. These findings showed outcomes important for inhibiting pyroptosis in clinical practice.

### 4.3 Necroptosis Treatment in Ischaemic Brain Injury

The key factor in necroptosis is RIPK3. MLKL is an important effector; therefore, inhibition of GSK872 via RIPK3 and necrosulfonamide in MLKL controls the progression of necroptosis. TRAF2 was also shown by Karl et al. [90] to inhibit TRAIL‐ and CD95L‐induced necroptosis. Li et al. [62] further studied TRAF2 and suggested that TRAF2 inhibited necroptosis by reducing the association between MLKL and RIP3. Tian et al. [91] proved that ponatinib combined with emricasan inhibited necroptosis after brain injury by inhibiting the combined pathways of caspase8 and RIPK3, showing importance as a reference for the treatment of necroptosis in clinical practice.

### 4.4 Ferroptosis in the Treatment of Cerebral Ischaemic Injury

Ferroptosis is caused by excessive accumulation of iron; therefore, targeting mechanisms and targeting free iron ions can affect its development. Deferoxamine, which targets iron ions, has been effective in targeting ferroptosis. Dixon et al. [39] demonstrated that liproxstatin‐1 was an inhibitor of ROS production; specifically, liproxstatin‐1 targeted the inhibition of erastin‐induced accumulation of cytosolic and lipid ROX and inhibition of GPX4 by selenium inhibited the progression of ferroptosis to a certain extent. In his experiments, Alim et al. [92] found that the ferroptosis inhibitor ferrostatin‐1 (Fer‐1) significantly reduced the degree of cerebral ischaemic injury in mice. Because ferroptosis is a relatively new field, despite research into its clinical applications, Yuki [93] found that sulfasalazine induced ferroptosis to treat pancreatic cancer. However, no research on specific drug effects on ferroptosis in brain injury has been reported.

### 4.5 Treatment of Cerebral Ischaemic Injury via Cuproptosis

Cuproptosis is similar to ferroptosis in that it is caused by the accumulation of metal ions, specifically copper ions. FDX1 is a key regulator of cuproptosis and an upstream regulator of protein–lipid acylation. The mitochondrial pyruvate carrier (MPC) inhibitor UK5099 and ETC complex I/III inhibitors (such as rotenone and antimycin A) attenuated ES‐induced cuproptosis [94]. FDX1 has been hypothesised to be an immune response predictor and prognostic biomarker [95], and another copper ionophore, disulfiram (DSF), has entered into phase II clinical trials for the treatment of malignant glioma [96]. ES is also in phase II clinical trials for melanoma [97]. The effectiveness of a cuproptosis inhibitor against cerebral ischaemia injury has not been verified and needs to be further studied.

Cerebral ischaemia injury is a complex pathophysiological event, and the aforementioned types of RCD are involved, mediated through different pathways and sites, leading to different forms of cell death. However, these RCD modalities all converge at a single axis; for example, the apoptosis pathways converge on caspase, and copper death pathways converge on FDX1. As long as we continue to explore the key sites and carry out targeted inhibition or induction at these sites, we can induce the necessary protective effect on cerebral ischaemic injury.

## 5 Discussion and Prospects

Brain ischaemia after cardiac arrest causes very serious and irreversible damage to the brain. In the protection against brain ischaemia after cardiac arrest, preventing and reducing cell death is a critical step. Currently, in the face of cerebral ischaemia injury after cardiac arrest, the treatment method is early reperfusion, but early reperfusion often leads to ischaemia–reperfusion injury. Whether it is possible to target the mechanism of cerebral ischaemic injury or to inhibit a certain type of injury to induce an RCD modality and thus protect the brain from cerebral ischaemic injury is still unknown.

The abovementioned RCD modalities, apoptosis, pyroptosis, necroptosis, ferroptosis and cuproptosis play important but different roles in different parts of the ischaemic brain. Pyroptosis, necroptosis and ferroptosis promote cell death [57, 98, 99, 100], and cuproptosis can promote brain nerve injury, but at the same time, as mentioned above, some studies have found that cuproptosis can inhibit nerve cell death [76, 101]. This contradictory result is also worth our follow‐up research, perhaps because the use of in vitro experiments cannot completely simulate the complex internal environment in the human body, so the opposite result is produced. For example, the pyroptosis, necrosis and apoptosis pathways in these studies may respond differently because of isolated experimental conditions that differ from clinical settings. In fact, the sites involved in these mechanisms have been studied extensively, and some drugs have been found to be effective or potential therapeutic targets. For example, hollow Prussian blue nanozymes protect nerves by inhibiting apoptosis [102], and NLRP3 may be a potential therapeutic target for pyroptosis [103]. Kaempferol can alleviate brain damage caused by ferroptosis by activating the Nrf2/SLC7A11/GPX4 signalling pathway [104]. Despite the recent publication of various reports on novel RCD activators and inhibitors, clinical trials evaluating the effects of novel RCD modulators are still in their infancy. At present, there are only two clinical studies on necrotic apoptosis (NCT04739618) and iron death (NCT05493800), and they are currently in the second phase, with no clear results, so exact results cannot be obtained. Although we have discussed these mechanisms separately in this review, in experiments and clinical practice, they are often found to be linked and influence each other. For example, when the caspase system was inhibited, the apoptotic system was inhibited, but necroptosis was found; therefore, it is possible that a similar compensatory system functions to ensure RCD. This possibility is worthy of discussion and research in the future. With the deepening of the research, some scholars have proposed that there may be crosstalk between different RCDs. Miao proposed [105] in his letter that ZBP1, a marker of cuproptosis‐related genes and necrotic apoptosis‐related genes, could be used as a risk score for predicting the prognosis of low‐grade glioma patients. Wei believed that elesclomol could affect both cuproptosis and ferroptosis. At present, studies have reported that elesclomol inhibits colorectal cancer by targeting ATP7A to manipulate cuproptosis and ferroptosis and have suggested elesclomol as a potential therapeutic agent for colorectal cancer [106], which is the best example of finding common ground for treatment by understanding the specific mechanisms of cells.

With known sites and effective ischaemia‐protecting drugs, some discoveries but no breakthroughs have been reported. In recent years, although research on various mechanisms of cerebral ischaemia injury has advanced, treatments for clinical applications are not available. At present, many drugs have been proven to be effective in inhibiting cerebral ischaemic injury in the laboratory [93, 103, 107], but no progress has been made in clinical practice. Although these drugs showed marked effects in animal experiments or in vitro cultured cells, the complex internal environment of the human body does not allow them to exert their effects; as a result, clinical application is not imminent. Of course, these cell death mechanisms may be targeted in the clinic, but applying a similar strategy to brain injury remains a challenge. Notably, drugs targeting cell death exert obvious effects on cancer and other diseases, but some of them have been used in clinical applications. Therefore, we need to continuously explore and discover the key factors underlying the effects of cell death on disease. Accordingly, these key factors can be leveraged for drug development and validation and applied as soon as possible to the treatment of cardiac arrest after brain ischaemia injury to prevent reperfusion injury, and thus attenuate cerebral ischaemia injury. It is believed that more high‐quality clinical trials on the mechanisms of ischaemic injury and RCD will be conducted after more cardiac arrest cases continue to emerge. In addition to laboratory research, some scholars explore databases and genes to guide the way for the experimental basis. For example, Qinlin Hua identified and verified genes related to cerebral ischaemia and copper milk disease. Eight central genes, including PDHA1, FDX1 and LIPT1, have been identified as potential new markers, which are expected to be used in clinical applications as specific biomarkers for the diagnosis, treatment and prognosis of cerebral ischaemia [108]. Therefore, we believe that soon, the mechanism of RCD will be better utilised to optimise the treatment of ischaemic injury after cardiac arrest.

## Author Contributions

**Zumin Chen:** writing – original draft (equal). **Shuangwei Wang:** writing – review and editing (equal). **Tian Shu:** methodology (equal). **Senlin Xia:** funding acquisition (equal). **Yanmei He:** data curation (supporting). **Yanhan Yang:** data curation (supporting).

## Conflicts of Interest

The authors declare no conflicts of interest.

## Data Availability

Data sharing is not applicable to this article, as no new data were created or analysed in this study.
